# The effect of weight loss and exercise on Health-Related Quality of Life (HRQOL) following Endoscopic Bariatric Therapies (EBT) for obesity

**DOI:** 10.1186/s12955-020-01359-3

**Published:** 2020-05-08

**Authors:** Gontrand Lopez-Nava, Ravishankar Asokkumar, T. Lacruz, A. Rull, L. Beltran, Inmaculada Bautista-Castaño

**Affiliations:** 1 Bariatric Endoscopy Unit, HM Sanchinarro Hospital, Calle de Oña, 10, 28050 Madrid, Spain; 2 Department of Gastroenterology and Hepatology, Singapore General Hospital, Singapore, Singapore; 3 Ciber of Obesity and Nutrition Pathophysiology (CIBEROBN), Instituto de Salud Carlos III, Madrid, Spain

## Abstract

**Introduction:**

Endoscopic bariatric therapies (EBT) have demonstrated to induce weight loss and improve comorbidities in obese patients. However, little is known about its impact on health-related quality of life (HRQOL) outcomes and physical activity status. This study aimed to evaluate the change in HRQOL and physical activity following EBT induced weight loss in obese patients.

**Methods:**

We approached 181 patients who underwent EBT in a standardized multidisciplinary follow-up program to participate in the study. We provided them two questionnaires-a) Short Form-36 health survey with the physical (PSC) and mental (MSC) summary component scores to capture generic HRQOL, and b) international physical activity questionnaire (IPAQ) for physical activity (PA). We administered the survey at baseline and at 9 months post-procedure. We expressed the procedure outcome as percentage total body weight loss (%TBWL). We expressed continuous variables as mean (SD) or median and categorical variables as percentages. We used non-parametric tests for comparison and performed multivariable linear regression analysis to identify factors associated with improvement in HRQOL.

**Results:**

The mean age was 42.2 (11.3) years, and the mean BMI was 38 (5.9)kg/m^2^. A majority of them were female (n-132, 73%). The EBT included intragastric balloons (n-136, 75%) and endoscopic sleeve gastroplasty (n-24, 25%). The mean %TBWL achieved after the intervention was 16.9 (9.7)%. We noticed a significant improvement in the median PSC (77.8 vs. 90.4, *p* < 0.001) and MSC (67 vs. 80.2, *p* < 0.001) scores after EBT. Similarly, we observed a significant positive change in physical activity compared to baseline (1606.2 vs. 2749 MET-minutes/week, *p* = < 0.001). Linear regression analysis showed an increase in %TBWL was associated with significant improvement in PSC (β = 0.193, *p* = 0.003) and MSC (β = 0.166, *p* = 0.02) scores of HRQOL, and likewise, increase in PA was independently associated with improvement in MSC (β = 0.192, *p* = 0.01). We did not find any difference in outcome based on gender or the type of intervention.

**Conclusion:**

EBT improves HRQOL in obese patients regardless of the type of intervention. The weight loss induced by EBT and the improvement in PA positively influence the health outcomes and quality of life.

## Introduction

Obesity is a complex disease and is associated with multiple comorbidities [1]. The rapid increase in the prevalence of obesity has significant implications on health care cost and policy development in Spain and worldwide [2, 3]. It is estimated that by 2030, one in 4 U. S adults will have severe obesity, and 14% of the annual healthcare expenditure would be related to obesity [4]. Data from the Framingham heart study showed that both females and males aged 40 years lost 7.1 years and 5.8 years due to obesity, respectively, and the reduction in life expectancy is higher in Class III obese patients [5]. Studies have established and demonstrated the quality of life years lost due to obesity has doubled from 1990 to 2008 [6].

Obesity and its associated complications produce a significant deterioration in health-related quality of life (HRQOL) [7, 8]. There is a social stigma surrounding obesity, and obese individuals are stereotyped to be lazy, lack self-discipline and are less motivated to adopt healthy behavior [9]. This, in addition to the clinical effects of obesity, results in emotional and mental distress, low self-esteem, impaired social life, reduced functioning, and decreased productivity [9, 10]. There is also a significant impact on physical functioning and mobility due to obesity [7]. Effective weight management strategies are required to assist obese patient to overcome the stigma and achieve an improved quality of life.

The available treatment options for obesity include diet and lifestyle therapy, pharmacotherapy, and bariatric surgery. The weight loss achieved with diet and lifestyle therapy or pharmacotherapy is only modest and rarely sustained beyond 6-months [11]. Conversely, bariatric surgery has been shown to induce and maintain significant weight loss over the long term [12]. Studies assessing HRQOL after bariatric surgery has demonstrated a positive change in physical and mental health at 3 and 6 years and improvement in physical activity (PA) [13–15]. However, despite its benefits, only a few patients opt for bariatric surgery because of its invasiveness, risk of complications, and cost [16].

To overcome this barrier, endoscopic bariatric treatment (EBT) options, including intragastric balloons (IGB) and endoscopic gastric volume reduction procedures like endoscopic sleeve gastroplasty (ESG), were developed with a premise to offer minimally invasive care at a lower cost and a shorter hospital stay [17]. Multiple studies have established its safety and efficacy at 12 and 24 months and showed improvement in obesity-related co-morbidities [18–20]. However, the quantum of weight loss achieved is lower as compared to bariatric surgery [20]. It is not known if endoscopic bariatric treatment solutions result in similar improvement in HRQOL and change in PA, as demonstrated with bariatric surgery. We hypothesized that the weight loss induced by EBT and the increase in PA after EBT might lead to an improvement in HRQOL. Our study aims to analyze the change in the HRQOL and PA using the validated short form 36 (SF-36) questionnaire and the international physical activity questionnaires (IPAQ) at baseline and 9 months in this unique population of patients receiving EBT. We compared our SF-36 results with the similarly assessed reference healthy Spanish population values [21]. We also examined the change in mental and physical summary components of HRQOL over time by age, sex, weight status, procedure type, and activity level.

## Methods

### Study design

We conducted a prospective study involving eligible patients who underwent EBT at HM Sanchinarro University Hospital, Madrid, Spain, between January 2015 to December 2016. We offered two questionnaires a) SF-36 survey (Spanish version) to measure the HRQOL, and b) International Physical Activity questionnaire to evaluate PA. The institutional review board approved the study. All authors had access to the study data and reviewed and approved the final manuscript.

### Participants

We invited 181 obese patients who were referred for EBT after a failed diet and lifestyle intervention to participate in the study. All agreed to take part and answered both the questionnaires at baseline and at 9 months post-procedure. We delivered the questionnaires during their regular follow-up visit. The inclusion criteria for EBT include a) Class 1–3 obesityand overweight patients with ≥2 comorbid illnesses (Type 2 diabetes mellitus, hypertension, lipid disorders, sleep apnea, osteoarthritis, non-alcoholic fatty liver disease, gastroesophageal reflux disease, and heart disease), and b) willingness to comply with multidisciplinary follow-up. We excluded patients with a) bleeding lesions in the stomach, b) peptic ulcer disease, c) large hiatal hernia (> 5 cm), d) malignancy, e) drug abuse and addiction, f) uncontrolled psychiatric illnesses, g) coagulopathy, h) severe systemic disease, and i) pregnancy.

The EBT was offered as a self-pay procedure, and all the patients paid up-front, including for the follow-up. The choice of procedure was based on patient preference. We collected information on their demographics, weight loss outcomes, and the procedure type. The weight and height were recorded using the Kern MPE (KERN & SOHN GmbH, Germany) weighing scale and stadiometer during each follow-up visit. We reported weight loss outcomes as %total body weight loss (%TBWL).

$$ \% TBWL=\left( Initial\ Weight- Final\ Weight\right)\ x\  100/\left( Initial\ Weight\right) $$

### Intervention

The EBTs we offered included IGBs (Orbera, Apollo Endosurgery, USA; ReShape Duo, Apollo Endosurgery, USA) and ESG (Overstitch, Apollo Endosurgery, USA). All the procedures were performed by a single expert endoscopist under anaesthesia support. The procedures were standardized, and there was no technical variation between the patients. We have presented our procedure technique in detail in our previous publications [20, 22, 23].

Post-procedure the patients were followed up at regular intervals by nutritionist, psychologist, and physiotherapist. Our unit has a standardized follow-up protocol for post-EBT patients. The energy requirements were calculated from the Harris-Benedict formula and, according to the type of physical activity, were decreased by about 2.6 MJ/day to induce an approximate loss of between 0.5 and 1 kg/week. In the first month, we maintained the patients on a strict liquid diet (4 weeks). We subsequently escalated the intake to semi-solid and solid food as tolerated. We designed our nutritional recommendation based on the Spanish society of nutrition guidelines [24]. We devised an individualized exercise plan (aerobic or resistance training- 30 min/day) depending on the capacity of the patients. We avoided exercised that increased the intra-abdominal pressure during the first month. At each visit, we discussed and encouraged compliance with diet and exercise recommendations.

### Questionnaires

#### Short form Survey-36 (SF-36)

The SF-36 is a well-validated questionnaire that measures the patients’ self-reported opinion on their physical and mental well-being [25]. It has eight domains of HRQOL: general health, physical functioning, role limitations due to physical health, energy/vitality, body pain, emotional well-being, role limitations due to emotional problems, and social functioning. Responses to each question within a domain are combined to generate a score from 0 to 100, where 100 indicates “good health.” The domains are calibrated and transformed into the physical component summary (PCS) and mental component summary (MCS), respectively. The SF-36 also includes a global health transition question (HTQ) that asks respondents to rate their general health compared with 1 year ago.

#### Reference Spanish population

We used the study by Lopez-Garcia et al. who prospectively evaluated the HRQOL in 6207 Caucasian non-institutionalized individuals aged > 18 years from Spain [21]. HRQOL was collected at baseline using the SF-12 survey, which is a reduced version of the SF-36 questionnaire. The SF-12 survey has been shown to correlate highly with the SF-36 questionnaire in obese and no-obese patients [26]. The study participants were classified into 3 groups based on their BMI and presence of one or more cardiometabolic comorbidities. For our study, we used the baseline HRQOL data of metabolically healthy normal weight (*n* = 1088) patients. The score for each of the 8 domains was available for comparison. We compared our baseline results to their healthy cohort.

#### The international physical activity questionnaires (IPAQ)

The validated IPAQ short version consists of 7 items that record physical activities performed by patients as a part of their daily life in the last 7 days [27]. It attempts to recall physical activity that was performed for at least 10 min at a time. Three characteristics of the physical activity were evaluated a) intensity of the activity (mild, moderate or vigorous), frequency (days/week), and duration (time/day). The weekly activity is recorded in Mets (Metabolic equivalent of Task). The reference Mets value used for calculation is: Walking-3.3 Mets; Moderate physical activity-4 Mets; Vigorous physical activity-8 Mets; The final value was derived by multiplying these reference values with the time (minutes) performed in a day and the numbers of days performed in a week.

$$ Total\  Met-\mathit{\min}/ week= Met-\mathit{\min}/ week\ walking+ Met-\mathit{\min}/ week\ moderate\  PA+ Met-\mathit{\min}/ week\ vigorous\  PA. $$

Based on the scores, we classified the patient into a) Inactive - < 600 Met-min/week, b) Minimally active ≥600 and < 3000 met-min/week, and c) Active ≥3000 Met-min/week.

### Outcome

The primary objective was to evaluate the changes in HRQOL and PA after EBT at 9 months. We examined the change in PSC and MSC scores over time by age, sex, weight status, procedure type, and activity level.

We also investigated the factors predictive for improvement in PSC and MSC of HRQOL.

### Statistical methods

We expressed continuous variables as mean (SD) or as median (range). We reported categorical variables as a percentage. For analysis, we categorized the variables based on a) gender (male/female), b) age (< 42 or ≥ 42 years), c) initial BMI (< 40 or ≥ 40 kg/m^2^), d) procedure type (IGB or ESG group), e) physical activity status (inactive or minimally active/active), and f) %TBWL (< 10% vs. 10–19.9% vs. ≥20%).

All variables were tested for normality using the Kolmogorov-Smirnov test. We used the Wilcoxon paired test for comparisons before and after the procedure. We used Mann Whitney or Kruskal Wallis test for comparison of HRQOL changes stratified by age, sex, initial BMI, procedure type, physical activity status, and %TBWL. We performed multivariable regression analysis to study the relationship between PSC and MSC scores and initial BMI, age, gender, % TBWL, changes in PA, and initial summary component values.

No information was available from previous studies on EBT to calculate the sample size based on improvement in the SF-36 domains. Accepting an alpha risk of 0.05 and a beta risk of 0.2 in a two-sided test, we estimated 157 subjects would be necessary to recognize a statistically significant difference of 5 or more points in the general health and physical function domain of SF-36 questionnaire and achieve a statistical power of 80%. The standard deviation was assumed to be 20, and the anticipated drop-out rate was 20% [28, 29]. We performed statistical analysis using SPSS Software19.0 (SPSS Inc., Chicago, Illinois, USA). *P* < 0.05 was considered significant.

## Results

### Participant characteristics

All 181 patients participated in the study. The mean age was 42.2 (11.2) years (range, 18–80), and a majority were female (*n* = 132, 73%). The mean initial BMI was 38 (6.0) kg/m^2^, and 32% were morbidly obese patients (*n* = 58). Most of the study participants received IGB (75%), followed by ESG (25%). The baseline characteristics of the patients are detailed in Table 1. None of the patients developed any serious adverse events after the procedure.

**Table 1:** Characteristics of study participants

Variables	Patients (***n*** = 181)
Mean Age, years	42.2 (11.3)
a) < 42 years	93 (51.4%)
b) ≥ 42 years	88 (48.6%)
Female	132 (73%)
Mean Initial weight, kg	106.7 (19.9)
Mean Initial BMI, Kg/m^2^	38 (6)
a) < 40 kg/m^2^	123 (68%)
b) ≥ 40 kg/m^2^	58 (32%)
Type of Procedure	
a) IGB	136 (75%)
b) ESG	45 (25%)
Baseline SF-36 (range)	
a) Median PSC score	77.1 (8.3–97.6)
b) Median MSC score	66.6 (10.7–98.5)
Baseline IPAQ	
a) Inactive	82 (45.3%)
b) Minimally active	63 (34.8%)
c) Active	36 (19.8%)
Mean %TBWL	16.9 (9.7)
a) TBWL < 10%	39 (21.5%)
b) TBWL 10–20%	80 (44.2%)
c) TBWL ≥20%	62 (34.3%)

Categorical variables express as n (%) and continuous variables as mean (SD) or median (range)

### Weight loss outcomes

All completed the follow-up. At 9 months, the %TBWL and BMI decline induced by IGB was 16.1 (7.8)% and 6.1 (3.3) kg/m^2^, respectively. Similar changes were induced by ESG [21 (9.3)%; 8.7 (4.4) kg/m^2^]. Almost 80% of the study participants demonstrated > 10% TBWL at the last follow-up appointment.

### Changes in HRQOL

**Fig. 1:**
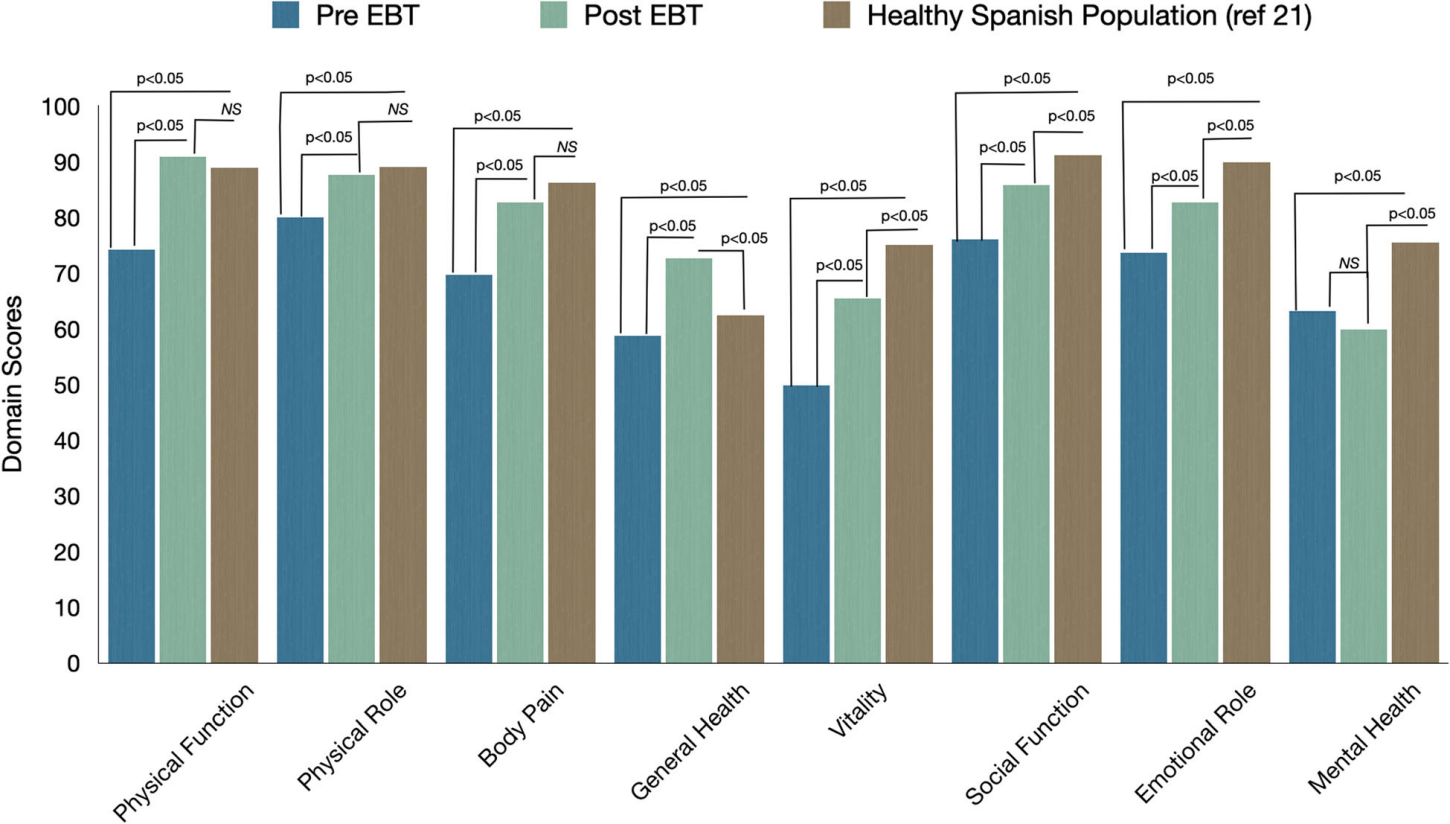
Comparison of SF-36 domains before and after EBT to the Spanish reference population. The HRQOL was significantly lower before EBT as compared to the healthy Spanish population. Post-EBT, the HRQOL improved significantly compared to baseline and was similar to the healthy reference population in physical function, physical role, and body pain domains

The baseline SF-36 score was significantly lower than the Spanish reference healthy population mean scores (Fig. 1). The median baseline PSC score was 77.1 (8.3–97.6), and the MSC score was 66.6 (10.7–98.5), respectively (Table 2). When stratified by their baseline characteristics, we observed higher PSC scores in patients with younger age (< 42 years), lower BMI (< 40 kg/m^2^), receiving IGB, and those who are minimally or physically active. The baseline MSC score was higher in men and was similar in all other groups (Table 3).

**Table 2:** Change in HRQOL after intervention compared to baseline in the study patients

	SF-36 Domains	Baseline		Post-Intervention		Mean Difference	***p***-value*
		Mean	Median	Mean	Median		
**PSC**	Physical function	74.2	80 (0–100)	90.9	95 (0–100)	16.7	< 0.001
	Physical role	78.7	100 (0–100)	87.7	100 (0–100)	9	0.003
	Body pain	69.4	70 (0–100)	82.7	90 (0–100)	13.3	< 0.001
	General health	58.4	60 (5–100)	72.7	75 (20–100)	14.3	< 0.001
**MSC**	Vitality	49.8	50 (0–100)	65.5	70 (0–100)	15.7	< 0.001
	Social function	74.7	75 (0–100)	85.8	100 (0–100)	11.1	< 0.001
	Emotional role	72.7	100 (0–100)	82.7	100 (0–100)	10	0.007
	Mental health	63.1	64 (12–100)	59.9	63 (0–80)	−3.2	0.11
	Health Transition	40.3	50 (0–100)	78.8	75 (0–100)	38.5	< 0.001

* the results were similar when compared using parametric test (mean) or non-parametric test (median). The values reported are from Wilcoxon paired test

**Table 3:** Comparison of HRQOL change based on patient characteristics at baseline and post intervention

	Physical Summary Component (mean)			Mental Summary Component (mean)		
	Baseline	Follow-up	Δ Change	Baseline	Follow-up	Δ Change
%TBWL						
< 10%	70.1	80	9.9	63.1	70.7	7.6
10–20%	74.5	86.7	12.1	64.5	74.3	9.8
≥ 20%	66.3	86.4	20.1	61.2	78.1	16.9
*p*-value	*0.06*		*0.02*	*0.72*		*0.07*
Gender						
Male	71.1	88.7	17.6	68.4	77.8	9.4
Female	70.6	83.8	13.5	61.0	73.5	12.5
*p*-value	*0.79*		*0.13*	*0.03*		*0.58*
Age						
< 42 years	76.8	88.7	11.9	65.0	76.0	11.0
≥ 42 years	65.0	81.7	16.7	61.1	73.4	12.3
*p*-value	*0.001*		*0.04*	*0.37*		*0.99*
Initial BMI						
< 40 kg/m^2^	75.1	85.9	10.8	63.4	75.0	11.6
≥ 40 kg/m^2^	61.6	83.5	21.9	62.2	73.9	11.7
*p*-value	*0.001*		*0.003*	*0.77*		*0.88*
Procedure Type						
IGB	73.3	87.0	13.7	64.8	77.6	12.8
ESG	63.1	79.6	16.5	57.8	64.1	6.3
*p*-value	*0.03*		*0.31*	*0.13*		*0.95*
Physical Activity						
Inactive	66.6	85.1	18.4	59.9	73.8	15.5
Active or minimally active	77.1	86.4	9.9	66.9	75.1	8.2
*p*-value	*0.001*		*< 0.001*	*0.23*		*0.49*

After the intervention, we found a significant improvement in the median PSC (77.1 vs. 90.4, *p* < 0.001) and MSC (66.6 vs. 80.7, *p* < 0.001) scores at 9 months. We observed a significant change in all domains except the mental health domain of the HRQOL. The domains that demonstrated the greatest improvements were physical function, vitality, and general health (Table 2). The health transition, as outlined in the survey, revealed a significant improvement after the intervention. We noticed significantly higher PSC score change in older patients (> 42 years), higher BMI (≥40 Kg/m^2^), physically inactive subjects, and those who achieved greater %TBWL (> 20%). The change in MSC scores were similar across the group, and a trend towards significant change was observed in patients with greater %TBWL (> 20%) (Table 3).

### Change in physical activity

At baseline, the majority of the patients were classified as inactive (45.3%, *n* = 82), followed by minimally active (34.8%, *n* = 63) and active (19.8%, *n* = 36). After the intervention, the mean Met-minute/week increased significantly (1606.2 vs. 2749.1, *p* < 0.001). More than 50% of the inactive patients at baseline became minimally active or active. At the last follow-up, 20.9% (*n* = 38) were inactive, 51.3% (*n* = 93) minimally active, and 27.6% (*n* = 50) were active. The mean hours per week of sitting decreased significantly (36.4 vs. 30.8 h, *p* < 0.01).

### Predictive factors for improvement in PSC and MSC

In linear regression analysis (Table 4), we found the factors that predicted change in PSC were increase in %TBWL [β = 0.193, 95%CI (0.068 to 0.780), *p* = 0.003] and lower initial PSC score [β = − 0.752, 95%CI (− 0.903 to − 0.627), *p* < 0.001].

**Table 4:** Multivariable linear regression analysis demonstrating factors associated with improvement in physical and mental summary components of SF-36

**Variable**	**Physical HRQOL (PSC change)**		
	**Coefficient β**	**95% Confidence Interval**	***p*-value**
Age	−0.07	−0.35 to 0.09	0.23
Sex	−0.08	−8.71 to 1.47	0.16
Initial BMI, Kg/m^2^	−0.04	−0.57 to 0.33	0.59
Mets change	0.10	0.0 to 0.001	0.09
%TBWL	0.19	0.17 to 0.78	0.003
Initial PSC score	−0.75	− 0.90 to − 0.63	< 0.001
**Variable**	**Mental HRQOL (MSC change)**		
	**Coefficient β**	**95% Confidence Interval**	***p*-value**
Age	−0.004	−0.29 to 0.27	0.95
Sex	−0.07	−10.52 to 3.57	0.33
Initial BMI, Kg/m^2^	−0.032	−0.69 to 0.44	0.67
Mets change	0.19	0.00 to 0.002	0.01
%TBWL	0.166	0.05 to 0.85	0.03
Initial MSC score	−0.62	−0.82 to 0.51	< 0.001

Similarly, the factors that predicted change in MSC were increase in %TBWL [β = 0.166, 95%CI (0.049 to 0.850), *P* = 0.028], increased PA measured by change in Met-min/week [β = 0.192, 95%CI (0.000 to 0.002), *p* = 0.010], and lower initial MSC score [β = − 0.622, 95%CI (− 0.823 to − 0.515), *p* < 0.001].

## Discussion

Our study shows that the quality of life of patients seeking EBT for obesity is lower than the healthy general population.. We demonstrated that EBT induces significant weight loss in obesity, and this may improve the HRQOL and physical activity status of patients irrespective of the procedure type.

Individual perception of the impact of a disease is a key deciding component for seeking treatment. Obesity with or without comorbidities has consistently demonstrated to cause deterioration in the health-related quality of life. The increase in weight over the years, and the rising BMI has shown to affect their physical function, decrease activity, and induce pain [30, 31]. Besides, obesity also impacts on the mental health of patients. Depression, mood disorders, and anxiety are frequently encountered in obese patients [9, 32]. Our study showed the baseline SF-36 scores of the study population were significantly lower to the normal healthy Spanish individuals [21]. The main objective of any treatment targeting obesity is to improve the quality of life and, if possible, revert to a normal healthy state.

Multiple studies have suggested a linear correlation between weight change and HRQOL [33, 34]. The modest weight loss induced by diet and lifestyle therapy alone may not be adequate to effect a change in HRQOL. The SHAPE-2 randomized control trial showed no significant improvement in the physical and mental domains of SF-36 after diet and exercise-induced weight loss [35].

On the contrary, both Roux-en-Y gastric bypass (RYBG) and laparoscopic sleeve gastrectomy (LSG) induced greater weight loss. They showed significant improvement in physical and mental summary component (SF-36) at 15 months and 24 months compared to baseline [36, 37]. Besides, they resulted in an overall improvement in physical activity at 2 years [15]. Despite the benefits, the invasive nature of bariatric surgery has certain disadvantages and has attracted only a small percentage of patients with obesity to seek surgery. There is a risk for the development of de-novo gastroesophageal reflux disease after LSG, which can impair the quality of life [37, 38]. These findings favor a need for less invasive effective therapy.

The results of our study showed EBT is effective in inducing weight loss. Studies evaluating comorbid improvement showed at least 5–10% weight loss is required to witness a change [39]. In our study, almost 80% of the patients achieved > 10% TBWL. Besides, the weight loss induced by EBT translated to improvement in HRQOL, as evidenced by the positive change in the physical and mental summary components similar to bariatric surgery. When compared with reference healthy population, the EBT patients had similar scores in physical function, physical role, body pain, and higher scores in general health after the intervention (Fig. 1). The IPAQ questionnaire showed a significant change in physical activity status compared to baseline. The observation raises a question if the change in PA could be a cause or consequence of weight loss after EBT. Fuller et al., in a randomized study, demonstrated that an increase in physical activity does not determine weight loss in a commercial weight loss program at 12 months [40]. However, physical activity plays a vital role in improving mental health. Chekroud et al., in a large cross-sectional study, showed improvement in mental health with a higher frequency of physical activity [41]. The increase in MSC scores after EBT lends support to this finding, and the exercise recommendation of our program is in close agreement with their recommendations. We believe the improved physical functioning with weight loss may have contributed to their increased physical activity, which in turn, led to an improvement in their mood and MSC. Additional assessment using the profile of mood state scale (POMS) may give us a better understanding to study the relationship between physical activity change and mental health [42].

Our study has several advantages. The available evidence on EBT has focused mainly on weight loss. Less emphasis and importance have been placed on the patient’s self-reported change in health following EBT. Our study is the first to address this clinically relevant question. The sample size was large, and the entire spectrum of obese patients was included in the study. Post-hoc analysis showed our study achieved a power of 85% with a sample size of 181. The procedure, nutrition, and the follow-up were standardized to avoid variation between the groups. However, there are certain limitations that are akin to a survey. Self-reported data for HRQOL might be impacted by recall bias. The patients were not blinded to the outcome measure, and there is a possibility of over-reporting of physical health and activity. There is also the problem of “present-state” bias when reporting health transition question as patients are inclined to judge their change in health status in relation to their present health state. The study is from a single center, and we evaluated HRQOL at 9 months after interventions. It is well known that weight regain is a significant problem with most bariatric therapies. Reassessment at a longer time interval with a larger sample is required to understand the efficacy of EBT. We utilized the generic SF-36 questionnaire to evaluate the HRQOL. Additional use of obesity specific questionnaires like the obesity-related problem scale may have addressed the psychosocial functional changes with EBT. We did not capture the role of socioeconomic determinants of health for all patients while measuring the change in HRQOL. However, EBT was offered as a self-pay procedure, and the included patients paid up-front, suggesting better economic well-being than the general population.

## Conclusions

Our study suggests that EBTs could be effective in inducing weight loss and improving the quality of life of patients with obesity significantly. The maximum benefit was observed in patients with higher initial BMI and physical inactivity at baseline. The tendency to induce such change using a minimally invasive procedure with a low complication rate may make EBT an attractive patient preferred treatment option. Further studies involving a control group and assessing in HRQOL at the long term are required.

## Data Availability

Please contact author for data requests.

## Abbreviations

HRQOL: Health-Related Quality Of Life
%TBWL: Percentage total body weight loss
BMI: Body mass index
EBT: Endoscopic bariatric therapies
IGB: Intragastric balloons
ESG: Endoscopic sleeve gastroplasty
SD: Standard deviation
PA: Physical activity
PSC: Physical summary component
MSC: Mental summary component
METS: Metabolic equivalent of task
SF-36: Medical outcome study 36-item health survey
IPAQ: International physical activity questionnaire
